# The relationship between psychiatric nurses’ perceived organizational support and job burnout: Mediating role of psychological capital

**DOI:** 10.3389/fpsyg.2023.1099687

**Published:** 2023-02-21

**Authors:** Yingxue Tang, Yingxuan Wang, Haiying Zhou, Juan Wang, Rui Zhang, Qinghua Lu

**Affiliations:** ^1^School of Nursing, Weifang Medical University, Weifang, China; ^2^Southampton Business School, University of Southampton, Southampton, United Kingdom; ^3^Department of Infection Management, Shandong Mental Health Center, Shandong University, Jinan, China; ^4^School of Public Health, Weifang Medical University, Weifang, China; ^5^Shandong Mental Health Center, Shandong University, Jinan, China

**Keywords:** job burnout, psychiatric nurses, perceived organizational support, psychological capital, intermediary effect

## Abstract

**Background:**

Psychiatric nurses need to keep close contact with patients suffering from mental illness. Because of the special nature of their profession, there is an increasing incidence of job burnout among psychiatric nurses.

**Aim:**

This study examined the relationship between psychiatric nurses’ perceived organizational support, job burnout, and psychological capital. It also investigated the mediating role of psychological capital in the relationship between their perceived organizational support and job burnout.

**Methods:**

A total of 916 psychiatric nurses were recruited from 6 grade-III mental facilities in Shandong Province using the stratified sampling approach. Their data were collected and examined using a general demographic data questionnaire, The Maslach Burnout Inventory, the Perceived Organizational Support Scale, and the Psychological Capital Questionnaire.

**Results:**

The total score of job burnout was 53.71 ± 16.37. Specifically, 73.69% of the nurses had moderate to severe emotional exhaustion, 76.75% had moderate to severe job burnout pertaining to depersonalization, and 98.80% had moderate to severe job burnout pertaining to personal accomplishment. Spearman’s correlation analysis showed that both psychological capital (*r* = −0.35, *p* < 0.01) and perceived organizational support (*r* = −0.31, *p* < 0.01) were adversely related to job burnout. Additionally, psychological capital somewhat mediated the relationship between perceived organizational support and job burnout. Its mediating impact accounted for 33.20% of the overall effect.

**Conclusion:**

This study’s participants had a moderate to severe level of job burnout. However, organizational support and psychological capital can be crucial in alleviating this problem among psychiatric nurses. Therefore, nursing managers and medical institutions should undertake timely and positive interventions to improve psychiatric nurses’ mental health and prevent job burnout. While exploring the impact of organizational support and psychological capital on job burnout, future studies should consider other effective influencing factors, and the relationship between the different factors should be explored in depth. This would provide a basis for developing a job burnout prevention mechanism.

## Introduction

According to Freudenberger (1974), job burnout is the consequent psychological state of employees feeling exhausted at work and being unable to achieve their goals. It consumes physical and mental energy, leading to exhaustion. According to a study (Tununu and Martin, 2020), job burnout is caused by long-term unresolved work stress and ineffective coping strategies. It affects workers’ psychology, physiology, and work. Previous studies showed that (Salvagioni et al., 2017) job burnout has a predictive effect in terms of cardiovascular and cerebrovascular diseases, hypermetabolic syndrome, pain, and other physical diseases as well as psychological conditions, such as depression and insomnia. It also often becomes an influencing factor in the case of these diseases/conditions. The incidence of job burnout is closely associated with one’s nature of work and work environment. Among various professions, teachers, doctors, and lawyers, to name a few, are the most prone to experiencing job burnout (Jin, 2007). A study showed (Dai et al., 2020) that 52.4% of medical workers suffer from job burnout, and those working in tertiary hospitals tend to suffer more from this problem. Job burnout is dominant in auxiliary jobs, and many researchers have found that the nursing staff is at a high risk of burnout (Melchior et al., 1997). Nurses play an extremely important role in the medical system, accounting for about 60% of the medical workforce (Soto-Rubio et al., 2020). Additionally, nurses tend to have the longest working days among all the medical and health professionals (Sousa et al., 2020), making them more vulnerable to job burnout. Moreover, burnout also has adverse consequences for work because it affects employees’ perceptions about job demands and resources (Rudman and Gustavsson, 2012), thus increasing employee absenteeism (Magtibay et al., 2017; Sarazine et al., 2021). The study by Ghavidel et al. (2019) also indicated that job burnout may lead to decreased work efficiency among nurses, increased absenteeism, and increased conflicts with patients or other staff members, leading to the possibility of decreased patient satisfaction. The nature of psychiatric nurses’ work differs from that of other medical workers, and it even differs from that of clinical nurses in other departments (Hughes and Umeh, 2005). Reportedly (Zhang and Li, 2020), the job burnout rate among psychiatric nurses in China is about 50%. Psychiatric nurses themselves are considered therapeutic tools for patients because they often need to continuously be in close contact with patients with mental disorders like psychosis, suicidal tendencies, and erratic behavior (Kurebayashi, 2019). In addition to being members of certain professional groups, psychiatric nurses play a crucial role in caring for patients with mental illnesses by being the long-term contacts for such patients (Wang et al., 2021). This intensifies their physical and mental burdens and makes them more prone to job burnout.

Boamah et al. (2017) designed a hypothetical model to identify the influencing factors of nurses’ job burnout. Their experiments revealed that nurses’ job burnout was related to a series of factors, such as structural empowerment, real leadership ability, and work-life interference. For organizational employees, fairness, supervisor support, organizational rewards, and favorable working conditions are related to building a strong sense of organizational support, and these are conducive to improving and increasing job satisfaction and positive emotions (Rhoades and Eisenberger, 2002; Ahmad et al., 2022). Some previous studies (Liu et al., 2018) indicated that organizational support can predict nurses’ turnover intention, and it plays a mediating role between their job satisfaction and job burnout. This implies that organizational support can affect nurses’ burnout to a certain extent. Eisenberger, an American psychologist, proposed the organizational support hypothesis (Eisenberger et al., 2001), from which the concept of perceived organizational support emerged. In this study, perceived organizational support was considered as nurses’ perceptions about their accomplishments being valued by their hospital as well as its concern for their welfare and physical and mental well-being; this imbibes a sense of obligation toward work and a belief of positive reciprocation at work (Puah et al., 2016). Psychiatric nurses’ department environment and their hospital’s administrative policies may affect their job burnout. When nurses’ sense of organizational support is high, their corresponding turnover intention and feeling of work pressure decreases (Li et al., 2020). It also simultaneously increases their sense of well-being and positive work behavior. Past research indicated that (Lowe et al., 2020) the job burnout of nurses is negatively correlated to their sense of organizational support. Therefore, increased social support and colleagues’ support would gradually decrease the burnout level of nurses. People with a high sense of organizational support have a sense of satisfaction with their organization that then translates into a sense of dependence on the organization. A high sense of organizational support can effectively relieve employees’ (psychiatric nurses) work pressure and reduce job burnout (Zheng and Wu, 2018). To add to this, Luthans et al. (2004) introduced the notion of psychological capital.

Psychological capital is formed in the process of individual growth and development (Guo et al., 2021). It is a four-part positive psychological state that includes self-efficacy, hope, tenacity, and optimism (Yan et al., 2020). Among these, self-efficacy is considered to be the most representative part of the aforementioned four aspects, and it is a regulating variable of work stress and job burnout (Molero Jurado et al., 2019). Moreover, psychological capital is an important part of organizational psychological behavior, and it is an available psychological resource that can be created and inculcated (Yang et al., 2020). An increasing number of people are becoming aware of the importance of psychological capital. Some studies showed that (Van der Heijden et al., 2019) nurses’ psychological capital is negatively related to job burnout, and it plays a mediating role in various influencing factors related to job burnout, such as pressure level (Zhu et al., 2021), job performance (An et al., 2020), emotional labor strategy (Peng et al., 2019), emotional intelligence (Gong et al., 2019), and so on. Thus, psychological capital can not only directly and negatively predict job burnout, but it can also be a moderating variable between job burnout and its various influencing factors. There is a positive correlation between psychological capital and organizational support (Liu et al., 2015), and some scholars believe that backed by the resource conservation theory and organizational support theory, organizational support can promote the positive development of employees by increasing psychological capital (Ho and Chan, 2022).

Consequentially, the following hypotheses were developed:

*H1:* Perceived organizational support is negatively correlated to job burnout.

*H2:* Psychological capital is negatively correlated to job burnout.

*H3:* Perceived organizational support is positively correlated to psychological capital.

According to the Job Demands-Resources (JD-R) model constructed by Demerouti et al. (2001), the interaction between job demands and job resources affects the development of burnout. Job demands are associated with certain psychological or physical costs. They correspond to certain life stressors like working hours, workload, work stress, and so on. Job resources are classified into external and internal resources. External resources include organizational and social resources, such as job control, support from colleagues or family, and so on. Internal resources include cognitive characteristics and action patterns. Bakker et al. (2005) proposed that job resources could buffer the impact of job demands on job burnout. In their study, when high job demands and low job resources appeared at the same time, the degree of employee burnout and depression was the highest. Therefore, this study explored the influence of internal and external job resources on job burnout, wherein psychiatric nurses’ perceived organizational support was the external resource, psychological capital was the internal resource, and job burnout was the result. Based on this, the following hypothesis was developed:

*H4:* Psychological capital moderates the relationship between perceived organizational support and job burnout.

This study explored the relationship between perceived organizational support, psychological capital, and job burnout. Additionally, it further examined the role and the intermediary mechanism of psychological capital (internal resource) in the relationship between perceived organizational support (external resource) and job burnout. Its findings can provide nursing managers and medical units with a theoretical basis for nursing management, improve the current situation regarding job burnout among psychiatric nurses, improve their work quality and efficiency, and improve the relationship between nurses and patients.

## Materials and methods

### Participants

**Figure 1:**
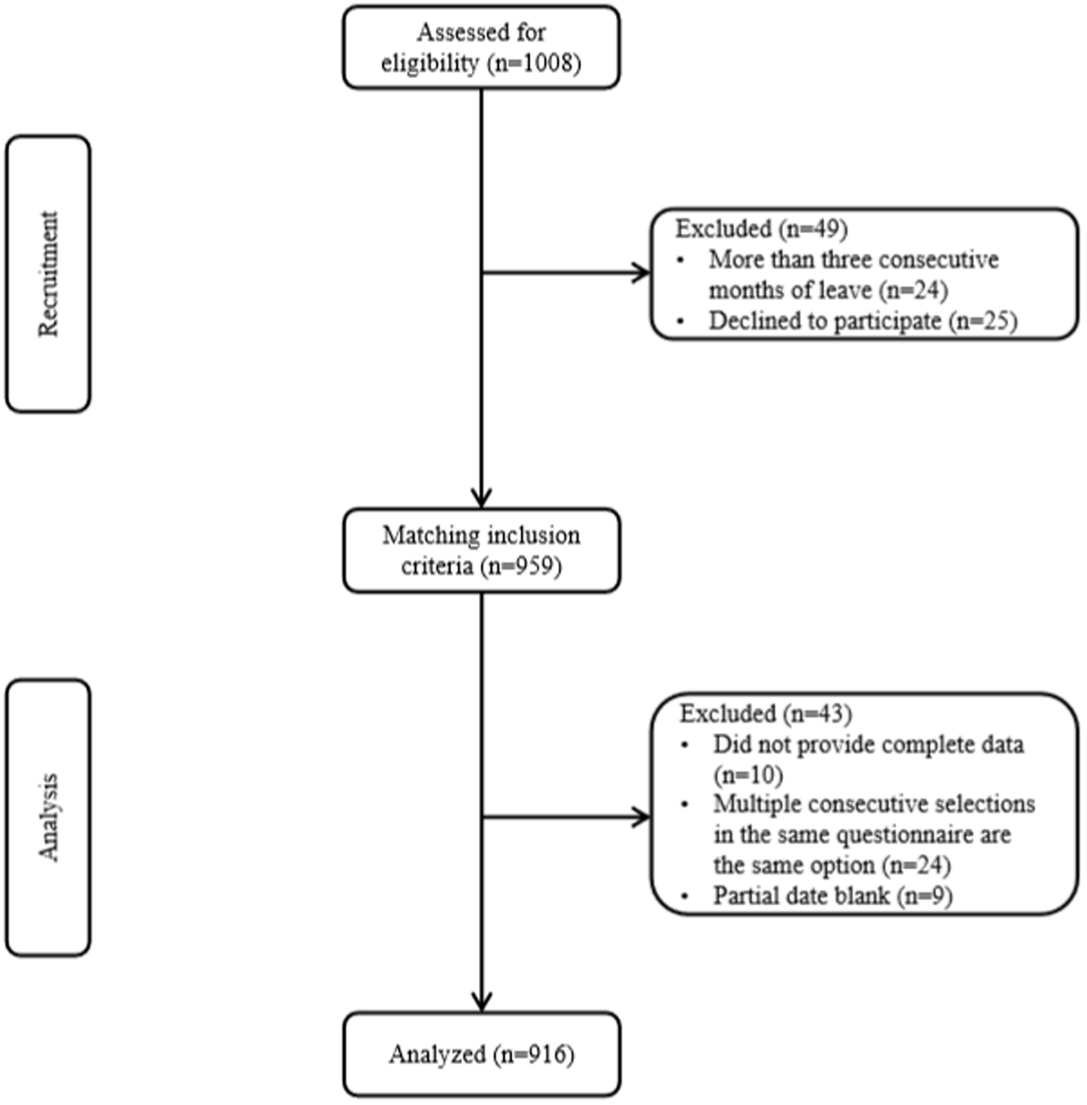
Flow chart showing study participants’ recruitment and study selection (*n* = 916, Shandong, China).

From August to September 2022, the stratified sampling method was adopted to stratify 17 prefecture-level cities in China’s Shandong province into 6 parts according to their geographical location: north, southwest, south, central, provincial capital area, and the peninsular area of Shandong Province. The random number table method was used to select one prefecture-level, tertiary, first-class psychiatric hospital for each part, with each hospital’s clinical front-line nurses as the research target. Inclusion criteria: (1) non-sex, marriage restricted nurses aged 18–60 years, (2) nurses who have worked for at least 1 year in hospitals specialized in mental illness, (3) nurses who have the national nurse practitioner registration qualification and are registered in the unit, (4) nurses working in clinical wards of psychiatric hospitals, and (5) psychiatric nurses who are on the job and voluntarily participated. Exclusion criteria: (1) regular nurses in non-psychiatric hospitals, including refresher nurses and trainee nurses in psychiatric hospitals, (2) nurses who have been on leave for more than 3 consecutive months, and (3) nurses who refused to participate in this study (Figure 1).

### Questionnaire collection procedure

The researcher provided appropriate training to the person in charge of each unit and explained the requirements for filling in the questionnaire. The person in charge distributed the questionnaire department-wise, using the unified guidance language. The respondents were given 20 min to fill in the questionnaire and returned it anonymously. A total of 1,008 questionnaires were distributed; 916 were valid, yielding an effective rate of 90.87%.

### Ethical considerations

The Ethics Committee of the Shandong Mental Health Center approved all the investigations involved in this study. The requirement of informed consent was carefully followed, and the information privacy of all the participants was ensured.

### Measures

#### General demographic data questionnaire

This questionnaire was intended to retrieve the basic information of all participants, including gender, age, marital status, educational level, professional title, working years, and monthly income.

#### Maslach Burnout Inventory

The Maslach Burnout Inventory (MBI) was designed to measure job burnout (Champion and Westbrook, 1984). The scale has 22 items under three aspects: emotional exhaustion (nine items), depersonalization (five items), and personal accomplishment (eight items). All items are graded on a scale of 0 to 6, with the scores for every element added together. Emotional exhaustion and depersonalization are positive scores, and the score ranges from 0 to 54 points and 0 to 30 points, respectively. Regarding depersonalization, a score of 2–5 is considered mild, 6–9 is considered moderate, and above 9 is considered severe. The higher the score in these two aspects, the more serious the burnout. Personal accomplishment is scored in reverse, with a score range of 0–48. A score of 39–45 is considered mild, 34–39 is considered moderate, and below 34 is considered severe. The lower the score, the more severe the burnout. The Cronbach’s coefficients for the subscales in this study were 0.887, 0.815, and 0.883, respectively.

#### Perceived organizational support scale

Perceived organizational support was measured using the simplified version of the Organizational Support Scale developed by Eisenberger and Stinglhamber (1986). It contains nine items, each of which is scored on a scale of 1 (completely disagree) to 7 (completely agree). Items 5 and 7 items are reversely scored, and the points from each item are added together to yield a total score that ranges between 9 and 63 points. The greater the total score, the more the organizational support. The scale’s Cronbach’s coefficient was 0.855 in this study.

#### Psychological Capital Questionnaire

The Psychological Capital Questionnaire (PCQ), compiled by Larson and Luthans (2006), is the most commonly used tool globally to measure psychological capital. Four characteristics of self-efficacy, hope, tenacity, and optimism are among the 24 items that make up the questionnaire. All items are scored on a scale of 1 (strongly disagree) to 6 (strongly agree). A score of 1–2 indicates a low level of psychological capital, 3–4 indicates a medium level of psychological capital, and above 4 indicates a high level of psychological capital, and the overall score falls between 24 and 144. Self-efficacy, hope, tenacity, and optimism all had Cronbach’s alpha values of 0.887, 0.899, 0.798, and 0.741 in this study. The scale’s Cronbach’s alpha value was 0.939.

### Statistical analysis

IBM SPSS Statistics for Windows, version 25.0 (IBM Armonk, NY, United States) statistical software was used for data entry and statistical analysis. Measurement data were expressed as mean and standard deviation, and enumeration data were expressed as frequency and percentage. The univariate analysis between sample characteristics and variables in general demography was evaluated by independent sample *t*-test and chi-squared test. Spearman’s correlation analysis and R4.1.2 were used to analyze the correlation between perceived organizational support, psychological capital, and job burnout. Hierarchical regression and Hayes’ Process Macro were used to investigate the mediating role of psychological capital in the relationship between perceived organizational support and job burnout. The test level *α* = 0.05, *p* < 0.05 was considered statistically significant.

## Results

### General information

A total of 916 psychiatric nurses returned valid, filled-in questionnaires (680 females, 74.24%; 236 males, 25.76%). The age of nurses was 32.75 ± 8.10 years (median 30.00 years; P2527.00, P7537.00); 76.42% of the total sample was married, and 23.58% was unmarried; 365 nurses had an educational level of junior college or below, and 551 were undergraduates and above, accounting for 39.85 and 60.15% of the total sample, respectively. The professional titles of the sample were divided into four categories: 310 nurses, 328 junior nurses, 230 senior nurses, and 48 associate superintendent nurses and above, accounting for 33.84, 35.81, 25.11, and 5.24% of the total sample, respectively. The number of working years was 10.19 ± 9.24 years (median 7.00 years; P253.00, P7515.00); 26.97% of the entire study sample, or 247 persons, drew a monthly income of less than 3,000 Yuan; 424 drew a monthly income of 3,000–5,000 Yuan, accounting for 46.29% of the total sample; and 26.75%, or 245 persons, drew a monthly income of more than 5,000 RMB (Table 1).

**Table 1:** Univariate analysis of psychiatric nurses’ job burnout, psychological capital, and perceived organizational support (*n* = 916, Shandong, China).

Variables	*n*	Constituent ratio (%)	Job burnout (M ± SD)	Psychological capital (M ± SD)	Perceived organizational support (M ± SD)
*Gender*					
Male	236	25.76	53.66 ± 17.63	101.15 ± 18.85	36.27 ± 10.65
Female	680	74.24	53.73 ± 15.92	99.59 ± 18.10	36.04 ± 10.28
*t*			−0.05	1.13	0.29
*p*			0.960	0.259	0.775
*Marital status*					
Single	700	76.42	53.26 ± 15.93	99.94 ± 17.80	35.64 ± 10.53
Married	216	23.58	55.16 ± 17.56	100.14 ± 19.86	37.60 ± 9.74
*t*			−1.49	−0.13	−2.43
*p*			0.136	0.894	0.015
*Educational background*					
College degree or below	365	39.85	54.35 ± 16.24	97.77 ± 19.22	36.20 ± 10.19
Bachelor’s degree or above	551	60.15	53.29 ± 16.45	101.46 ± 17.52	36.03 ± 10.50
*t*			0.96	−3.01	0.24
*p*			0.337	0.003	0.808
*Age (in years)*					
≤30	502	54.80	53.71 ± 16.38	99.26 ± 18.53	37.05 ± 9.61*
31–40	239	26.09	56.68 ± 15.85	98.51 ± 18.24	34.71 ± 10.57
41–50	137	14.96	48.78 ± 16.01*	103.25 ± 17.00	36.05 ± 11.44
>50	38	4.15	52.82 ± 17.15	107.24 ± 17.71*	32.53 ± 13.15
*F*			6.93	4.27	4.39
*p*			0.000	0.005	0.004
*Professional title*					
Nurse	310	33.84	52.60 ± 16.34	99.63 ± 18.65	37.65 ± 9.09
Junior nurse	328	35.81	55.58 ± 16.44*	98.74 ± 17.82	35.74 ± 10.30
Senior nurse	230	25.11	53.43 ± 16.41	101.27 ± 18.30	34.11 ± 11.55*
Associate superintendent nurse	48	5.24	49.48 ± 14.80	104.71 ± 18.66	38.10 ± 11.12
*F*			3.01	1.99	5.94
*p*			0.029	0.113	0.001
*Number of working years*					
≤10 years	615	67.14	54.23 ± 16.43	99.31 ± 18.34	36.80 ± 9.76
11–20 years	142	15.50	55.39 ± 15.36	99.70 ± 18.64	34.94 ± 10.56
21–30 years	124	13.54	49.56 ± 16.46*	101.29 ± 17.75	34.56 ± 12.03
>30 years	35	3.82	52.54 ± 17.18	108.49 ± 16.30*	34.03 ± 12.67
*F*			3.45	3.04	2.92
*p*			0.016	0.028	0.033
*Monthly income*					
<3,000yuan	247	26.97	52.57 ± 15.89	97.18 ± 18.61*	35.89 ± 10.02
3,000–5,000yuan	424	46.29	54.23 ± 15.67	99.08 ± 17.71	35.88 ± 9.60
>5,000yuan	245	26.75	53.96 ± 17.97	104.39 ± 18.28	36.69 ± 11.92
*F*			0.84	10.74	0.54
*p*			0.432	0.000	0.583

Age: Compared with “31–40,” *p < 0.05; Professional title: Compared with “Nurse” *p < 0.05; M, mean; Number of working years: Compared with “≤10 years,” *p < 0.05; Monthly income: Compared with “>5,000 Yuan,” *p < 0.05; SD, standard deviation; *t*, values of *t*-test; *F*, values of ANOVA; *Post Hoc* Multiple Comparisons: Scheffe.

### Psychiatric nurses’ perspective on job burnout, organizational support, and psychological capital

The total score of job burnout of psychiatric nurses was 53.71 ± 16.37, and the score of emotional exhaustion was 24.16 ± 9.98, of depersonalization was 10.27 ± 5.87, and of personal accomplishment was 19.28 ± 7.26. The scores of these three aspects were divided into “mild,” “moderate,” and “severe.” A total of 675 nurses with moderate and severe emotional exhaustion accounted for about 73.69% of the total study sample, 703 nurses with moderate and severe depersonalization accounted for about 76.75%, and 905 nurses with moderate and severe sense of personal accomplishment accounted for about 98.80% of the total study sample. The total score of perceived organizational support was 36.10 ± 10.37. The total score of psychological capital was 99.99 ± 18.30, of which the “self-efficacy” score was 25.36 ± 5.43, the “hope” score was 25.54 ± 5.49, the “tenacity” score was 24.15 ± 4.83, and the “optimism” score was 24.94 ± 5.30.

### Comparison of scores of psychiatric nurses’ job burnout, perceived organizational support, and psychological capital with different characteristics

In terms of job burnout, there was a major difference based on age (*F* = 6.93, *p* = 0.000), with nurses aged 41–50 years scoring lower than other age groups (*p* < 0.05). The scores of psychiatric nurses aged less than 30 years (*F* = 4.39, *p* = 0.004) were higher than those of other age groups (*p* < 0.05). The scores of psychiatric nurses who were over 51 years old (*F* = 4.27, *p* = 0.005) were higher than those of others (*p* < 0.05). There are significant professional differences among nurses in terms of their designation, i.e., junior nurse, senior nurse, associate superintendent nurse (*F* = 3.01, *p* = 0.029), and so on. Junior nurses had the highest burnout scores (*p* < 0.05). The scores of senior nurses (*F* = 5.94, *p* = 0.001) were lower than that of the nurses with other professional titles (*p* < 0.05). There was also a big variation based on the number of working years (*F* = 3.45, *p* = 0.016). Psychiatric nurses who worked 21–30 years had the lowest scores (*p* < 0.05). Those with more than 31 working years (*F* = 3.04, *p* = 0.028) scored higher than the others (*p* < 0.05). The perceived organizational support among married psychiatric nurses was higher than that among unmarried nurses (*t* = −2.43, *p* = 0.015). The psychological capital scores of psychiatric nurses with a higher education level were higher (*t* = −3.01, *p* = 0.003). The scores of psychiatric nurses with monthly income (*F* = 10.74, *p* = 0.000) less than or equal to 3,000 Yuan were lower than the others (*p* < 0.05). Table 1 summarizes the results.

### Correlation analysis between job burnout, perceived organizational support, and psychological capital

**Figure 2:**
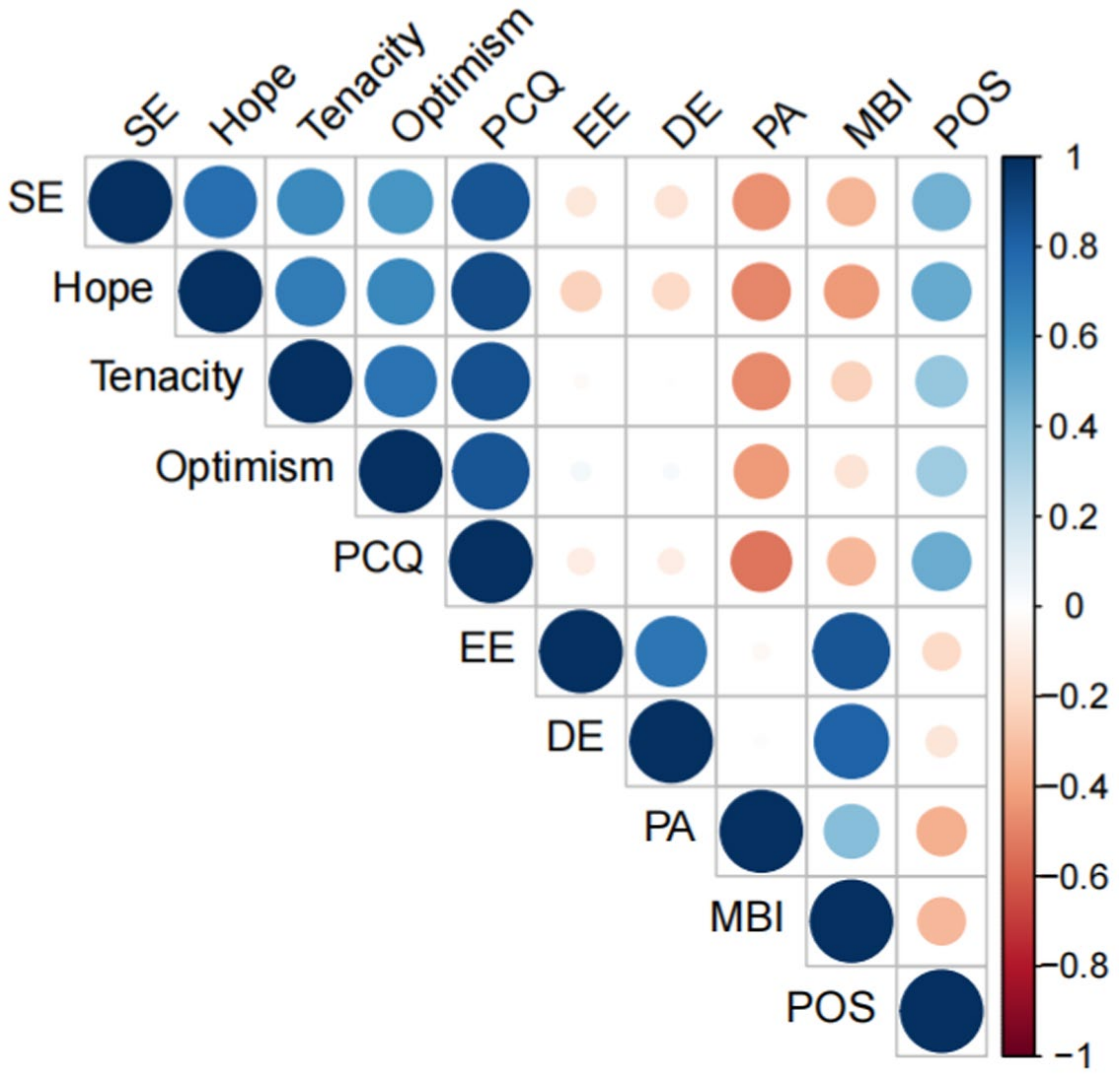
Heat map of psychiatric nurses’ job burnout, psychological capital, and perceived organizational support (*n* = 916, Shandong, China). PCQ, Psychological Capital; POS, Perceived Organizational Support; MBI, Job Burnout; SE, Self-efficacy; EE, Emotional Exhaustion; DE, Depersonalization; PA, Personal Accomplishment.

The Spearman’s Rho analysis revealed a significant relationship between job burnout, perceived organizational support, and psychological capital. Psychiatric nurses’ perceived organizational support was negatively correlated with their overall job burnout score (*r* = −0.31, *p* < 0.01) as well as the three aspects of emotional exhaustion (*r* = −0.24, *p* < 0.01), depersonalization (*r* = −0.18, *p* < 0.01), and personal accomplishment (*r* = −0.37, *p* < 0.01). The total scores of job burnout and psychological capital (*r =* −0.35, *p* < 0.01) and its four aspects of self-efficacy (*r* = −0.35, *p* < 0.01), hope (*r* = −0.41, *p* < 0.01), tenacity (*r* = −0.24, *p* < 0.01), and optimism (*r* = −0.17, *p* < 0.01) were all negatively correlated. The perceived organizational support was associated with self-efficacy (*r* = 0.83, *p* < 0.01), hope (*r* = 0.87, *p* < 0.01), tenacity (*r* = 0.84, *p* < 0.01), and optimism (*r* = 0.82, *p* < 0.01) as well as the overall psychological capital score (*r* = 0.46, *p* < 0.01). Table 2 shows the correlation analysis between the other aspects. To more clearly describe the correlation between the indicators, we plotted a correlation heat map using R 4.1.2, as illustrated in Figure 2.

**Table 2:** Correlation between psychiatric nurses’ perceived organizational support, job burnout, and psychological capital (*n* = 916, Shandong, China).

Variables	1	2	3	4	5	6	7	8	9	10
1. Perceived organizational support	1									
2. Self-efficacy	0.44**	1								
3. Hope	0.47**	0.71**	1							
4. Tenacity	0.34**	0.57**	0.65**	1						
5. Optimism	0.33**	0.53**	0.59**	0.69**	1					
6. Psychological capital	0.46**	0.83**	0.87**	0.84**	0.82**	1				
7. Emotional exhaustion	−0.24**	−0.18**	−0.26**	−0.08*	−0.03	−0.17**	1			
8. Depersonalization	−0.18**	−0.24**	−0.26**	−0.09**	−0.05	−0.19**	0.69**	1		
9. Personal accomplishment	−0.37**	−0.46**	−0.49**	−0.45**	−0.37**	−0.52**	0.08*	0.18**	1	
10. Job burnout	−0.31**	−0.35**	−0.41**	−0.24**	−0.17**	−0.35**	0.86**	0.83**	0.45**	1

** *p* < 0.01;

* *p* < 0.05.

### Analyses of psychological capital’s mediating role in the relationship between psychiatric nurses’ perceived organizational support and job burnout

**Figure 3:**
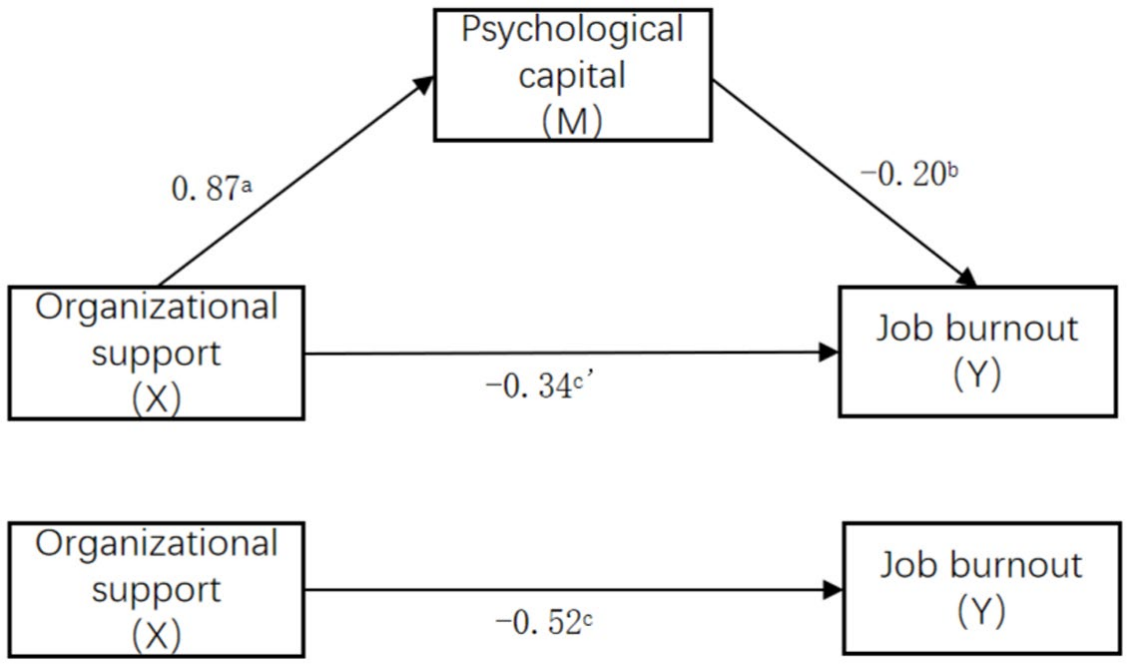
The intermediary path of psychological capital between perceived organizational support and job burnout (*n* = 916, Shandong, China). a: direct effect of X on M; c’: direct effect of X on Y; a* b: indirect effect of X on Y; c: Total effect; *p* < 0.01.

It was assumed that psychological capital has a mediating effect between psychiatric nurses’ perceived organizational support and job burnout, and the mediating path is shown in Figure 3. However, its mediating effect was tested based on the analysis regarding the same by Du et al. (2014). Three regressions were conducted with general demographic data as the control variable, job burnout as the dependent variable (Y), perceived organizational support as the independent variable (X), and psychological capital as the mediating variable (M) (Table 3). For the first time, general demographic data were added to the first layer, and perceived organizational support was added to the second layer to explore the predictive effect of independent variables on the dependent variable, namely job burnout. In the second regression, control variables were added to the first layer, and independent variables were added to the second layer to explore the predictive effect of independent variables on the mediating variable, namely psychological capital. In the third regression, the control variables were added to the first layer, independent variables were added to the second layer, and control variables were added to the third layer to explore the predictive effect of mediation variables on the dependent variable after controlling the first and second variables.

**Table 3:** Mediating effect analysis of psychiatric nurses’ psychological capital of psychiatric nurses (*n* = 916, Shandong, China).

Variables	Job burnout (B)	Psychological capital (B)	Job burnout (B)
Gender	−0.42	2.51	0.07
Marital status	0.66	−2.09	0.26
Education background	−2.60	0.75	−2.45
Professional title	2.42	1.15	2.64
Number of working years	−1.75	2.77	−1.21
Monthly income	0.97	−1.45	0.69
Age (in years)	2.56	2.02	2.95
Perceived organizational support	−0.55	0.88	−0.37
Psychological capital	–	–	−0.20
*F*	121.55	305.79	37.24
*R* ^2^	0.13	0.28	0.17
△*R*^2^	0.12	0.27	0.16
*p*	0.000	0.000	0.000

*B*, unstandardized regression coefficient; *R*^2^, coefficient of determination; △*R*^2^, adjusted coefficient of determination; *F*, values of ANOVA.

The bootstrap technique was utilized to examine the mediating role of psychological capital based on hierarchical regression, utilizing the process 3.2 plug-in suggested by Hayes (2014). The “model number” was set to 4, the “number of bootstrap samples” was set to 5,000, and the confidence interval was set to “significant.” The “95% confidence interval (*CI*)” in the results did not include 0, i.e., *p* < 0.01, indicating that the mediating effect was statistically significant. The findings indicated that psychological capital may play a role in the relationship between perceived organizational support and job burnout because the 95% CI of its moderating mechanism was −0.23 to −0.11, and the *p* values within the interval of 0 were less than 0.01. Job burnout played a partial mediating role, and the mediating effect was statistically significant, accounting for 33.20% of the total effect. Table 4 shows the specific data of the bootstrap test. It also shows the total effect, direct effect, and indirect effect of each pathway.

**Table 4:** Verifying the bootstrapping mediation effect (*n* = 916, Shandong, China).

Psychological capital	B	Bootstrap SE	Bootstrap 95%CI	
			LLCI	ULCI
Total effect	−0.52	0.05	−0.66	−0.42
Direct effect	−0.34	0.06	−0.45	−0.23
Indirect effect	−0.17	0.03	−0.23	−0.11

*B*, unstandardized regression coefficient; SE, standard error; CI, confidence interval.

## Discussion

### Demographic analysis of job burnout, organizational support, and psychological capital in psychiatric nurses

In this study, the total score of job burnout was (53.71 ± 16.37), and most of the 916 nurses had different degrees of job burnout. Regarding the three aspects, moderate and severe cases accounted for more than 70%. The findings of Acea-López et al. (2021) and Wang et al. (2019) pertaining to nurses’ job burnout differ from ours. The degree of job burnout in this study was higher than in other studies (Khamisa et al., 2015; Wang et al., 2019; Acea-López et al., 2021), as this study’s participants evidently had higher job burnout. The incidence of moderate to severe cases was also higher than in other studies. This may be because of the different participants or the special care objective of the relevant psychiatric departments. This study included only psychiatric specialist nurses. The comparative study’s participants included nurses from various departments in general hospitals as well as nursing staff in nursing homes, which may be one of the reasons for the different findings. Psychiatric patients often exhibit abnormal behavior and thinking, and they are impulsive and difficult to control. In a Saudi Arabian study on mental health facilities (Alenezi et al., 2019), the detection rates of above-moderate burnout among caregivers in the control and intervention groups at baseline were approximately the same as in this study, indicating that psychiatric nurses are more likely to experience burnout. There are many factors underlying this.

In this study, there were significant differences in the scores of job burnout based on professional titles, working years, and age. Many demographic factors can also lead to job burnout among nurses. Psychiatric nurses aged 41–50 years and who have worked for 21–30 years may be in the same situation and yet have the lowest job burnout score, which may be related to their being married as well as their family and job proficiency. Psychiatric nurses at this stage are familiar with the hospital’s working environment, various working modes, and various emergencies they may suffer from. They have a thorough understanding of the causes, manifestations, and characteristics of various mental diseases, and hence, they can cope with various emergencies better. Needless to say, older, experienced psychiatric nurses are less stressed than new nurses, and they are less likely to suffer from job burnout. Okwaraji and Aguwa (2014) also claimed that young nurses were more prone to job burnout compared to older nurses, which is related to their lesser work experience and lack of sufficient psychological flexibility to cope with the many challenges at work. In a study by Kim and Kweon (2020), nurses with more work experience showed lower burnout.

There were significant differences in perceived organizational support scores of psychiatric nurses of different ages and professional titles. Young nurses may have heavy workload, low pay, and no sense of belonging to the work unit, which will lead to a lower score of organizational support for young nurses than for older nurses (Lin et al., 2020). The psychological capital scores of psychiatric nurses with different ages, working years, and monthly income are significantly different, which is consistent with the findings of Zhao et al. (2022). Older psychiatric nurses with longer working hours and higher monthly income had higher levels of psychological capital. Studies have shown (Li et al., 2022) that psychological capital is closely related to job performance and self-confidence. Psychiatric nurses, with increasing age and working years, continue to increase their knowledge reserve. This enhances their ability to self-regulate and adapt to the environment, thus increasing the level of their psychological quality and psychological capital as compared to young nurses.

### The relationship between job burnout, perceived organizational support, and psychological capital

The results revealed a correlation between perceived organizational support, job burnout, and psychological capital. There was a specifically significant correlation between perceived organizational support and job burnout. Our study showed that the higher the perceived organizational support, the lower the job burnout, which is consistent with most previous research results (Eisenberger et al., 2001; Wu et al., 2018; Yu et al., 2021). From the perspective of perceived organizational support, reducing job burnout of psychiatric nurses requires nursing managers and hospitals to strengthen the organizational support mechanism for psychiatric nurses. Socio-environmental factors, such as the hospital’s working environment and the interpersonal communication between nurses affects their perceived organizational support and affecting the speed of their mental recovery. This could be connected to how nurses feel at work. When psychiatric nurses realize their value at work and sense the environmental or other forms of support from colleagues (Lowe et al., 2020), departments, and even the hospitals, their psychological or physical pressure at work lessens, thus reducing job burnout. China’s work environment is characterized by a collectivist culture, hierarchical structure, and paternalistic leadership (Zheng and Wu, 2018). Thus, a low job burnout caused by high perceived organizational support suggests that in the work environment of psychiatric nurses, the head nurse or other managers provide them with a high degree of attention, leadership guidance, and supervision.

Additionally, this study also found that psychological capital was negatively correlated to job burnout and positively correlated to perceived organizational support. It can be predicted that psychiatric nurses’ increased psychological capital and perceived organizational support would decrease their job burnout. The finding that psychological capital negatively affects job burnout is in line with other scholars’ research findings (Luthans and Youssef, 2004; Peng et al., 2013). Psychological capital is a positive psychological characteristic, which is composed of positive personal resources. Studies have shown that (Qiu et al., 2019) this positive psychological state can improve the ability of psychiatric nurses to overcome difficulties and challenges that arise during clinical work and strengthen their willingness to achieve their ideal goals. The four psychological capital-related factors of self-efficacy, hope, tenacity, and optimism entail plasticity, which can affect the psychological status and work ability of psychiatric nurses. Therefore, hospitals can enhance the psychological capital of psychiatric nurses through a lot of training and learning (Lee and Kim, 2017). When psychiatric nurses encounter emergencies at work, those with high psychological capital would habitually think and deal with problems from a positive perspective. They would be good at introspecting from a position of failure and summarizing experience from success. When the nursing staff from some difficulties to escape, high psychological capital can use a shorter time to adjust their state, continue to use a positive attitude, full of mental state work. Owing to challenging problems, nurses with high psychological capital would not give up easily or feel depressed, and they may even insist on efficient work engagement. Therefore, high psychological capital makes psychiatric nurses more likely to calmly face difficulties at work, thus reducing the occurrence of job burnout.

### Analyses of psychological capital’s mediating role in the relationship between perceived organizational support and job burnout

This study further verified the JD-R model of job burnout, which implies that perceived organizational support is an external work resource. Considering that individual work needs are fixed and cannot be freely changed, effective organizational support can help individuals cope with the negative impact of the environment and achieve their personal goals. Moreover, psychological capital, as an internal work resource, is relatively stable. It is also an effective protective factor for individuals in terms of coping with the negative effects of work, thus reducing job burnout. Perceived organizational support explained 12.4% of the variance of job burnout in the participating psychiatric nurses, and the model including perceived organizational support and psychological capital explained 15.7% of the variance of job burnout. Further, from the perspective of intermediary effect verification, the indirect effect of psychological capital accounted for 33.20% of the total effect. The perceived organizational support can not only directly affect the job burnout of psychiatric nurses, but also indirectly affect job burnout through psychological capital. The social cognitive theory believes that one’s environment can affect their internal cognition and thus affect their response to the environment (Xu et al., 2017). As an adjustable positive psychological resource, psychological capital has a great impact on the sense of organizational support and job burnout among psychiatric nurses. Their perceived organizational support is affected by many factors. A hospital-registered nurse (Trybou et al., 2014) expresses hope that the facility can do more to improve the conditions in which the nurses work and also hopes that nursing managers formulate policies to improve their sense of organizational support. People with a higher level of perceived organizational support are more likely to accept the reinforcement of psychological capital and are more likely to strengthen the construction of a positive psychological state (Ren et al., 2021), thus reducing the job burnout. Studies showed that (Khalid et al., 2020) psychological capital is positively related to nurses’ job satisfaction, job performance, quality of life, and organizational commitment. This requires nursing managers and medical institutions to make corresponding changes, such as improving nurses’ salary, timely fulfilling all kinds of commitments to nurses’ work and life, and improving nurses’ job satisfaction. Research also showed that (Peng et al., 2013) people with high psychological capital are more likely to receive organizational support, are more willing to contribute to the organization, and show less burnout (Tian and Xie, 2010). Therefore, psychological capital is a mediator of perceived organizational support and affects psychiatric nurses’ burnout. The current study is meaningful because it emphasized the importance of psychological capital in regulating the job burnout of psychiatric nurses.

To prevent psychiatric nurses from developing job burnout or to reduce their existing job burnout, nursing managers should strengthen communication with nurses and identify their physical or psychological abnormalities on time. An overall symposium every week should be conducted and psychological treatment methods, such as interpersonal psychotherapy and group therapy should be adopted. Balint group activities (Ren and Pang, 2018) can also be introduced in regular meetings to reduce nurses’ anxiety, depression, emotional exhaustion, and other problems. Psychiatric nurses can be trained in various skills that would help them improve professionally, provide high-quality nursing to patients, promote the rehabilitation of patients, strengthen their sense of personal accomplishment in nursing, and reduce job burnout. Additionally, it is necessary to strengthen the training of nursing managers, improve their management and leadership ability, and cultivate the ability of diversified communication between nursing managers and psychiatric nurses, which can make nurses feel like they are cared for by the hospital and leadership. This, in turn, would improve their perceived organizational support. Thus, to summarize, nursing managers and medical institutions should try to improve psychiatric nurses’ perceived organizational support, increase their positive psychological capital, and reduce their job burnout through various direct and indirect ways.

## Limitations

This study has some limitations. First, the study included a sample of psychiatric nurses from only one province in China. It would be useful to replicate this study among psychiatric nurses across other provinces to compare findings and improve generalizability. Second, the study used self-reported questionnaires that nurses filled in to assess job burnout. This entails a lack of objectivity. In the future, researchers may consider adding other people’s evaluations to enhance objectivity. Third, there may be limitations in the process of questionnaire delivery; the method should be improved in future studies and the questionnaire should be issued by the researchers themselves. Fourth, it was a transversal study, making it difficult to establish causal relationships between the variables. Future research should also examine reverse relationships between the variables. In addition to the factors studied, other aspects should also be considered in a national longitudinal study on perceived organizational support, job burnout, and psychological capital of psychiatric nurses in the future.

## Practical implications

This study randomly selected six grade-III mental facilities in Shandong Province and investigated the relationship between psychiatric nurses’ perceived organizational support, job burnout, and psychological capital. In cases of a long-term pressure-inducing work environment, people are unable to effectively relieve and release their stress. Hence, they easily fall prey to fatigue and emotional exhaustion, especially in the caregiving industry. This study has practical implications for clinical psychiatric nurses, nursing managers, and medical institutions/governments in the context of psychiatric hospitals. First, for psychiatric nurses, this study entirely identified the influencing factors of psychiatric nurses’ job burnout and provided the relevant basis for their self-intervention regarding job burnout. Second, for nursing managers and hospitals, this study could help them identify the underlying factors of job burnout among psychiatric nurses. This, in turn, would help managers formulate targeted interventions to reduce burnout according to the given situation. Finally, this study established that perceived organizational support indirectly affects job burnout through psychological capital. Hence, the government can formulate some favorable policies to improve the overall environment of medical and health institutions.

## Conclusion

For the three aspects of emotional exhaustion, depersonalization, and personal accomplishment on the job burnout scale, the detection rate of moderate to severe job burnout was above 70%. Specifically, the moderate to severe rate of emotional exhaustion was 73.69%, of depersonalization was 76.75%, and of personal accomplishment was 98.80%. There were differences based on age, job title, and the number of working years. Psychiatric nurses have a high incidence of job burnout, which should be paid more attention to. There were significant differences in the perceived organizational support scores of the psychiatric nurses across different age groups and professional titles. Their psychological capital differed significantly according to age, the number of working years, and monthly income.

Job burnout, perceived organizational support, and psychological capital of psychiatric nurses were correlated. Psychological capital and perceived organizational support were adversely associated with job burnout. There was a positive correlation between psychological capital and perceived organizational support.

Psychological capital of psychiatric nurses somewhat mediated the relationship between their perceived organizational support and job burnout, accounting for 33.20%.

Recent studies found that personality dimensions (Khosravi, 2021) and high novelty-seeking (Khosravi et al., 2020) also have a significant impact on job burnout. For example, higher novelty-seeking scores are associated with higher levels of burnout. Future research should therefore devote more attention to this problem and study various factors that may affect job burnout in depth to effectively prevent and reduce job burnout across various industries.

## Data availability statement

The original contributions presented in the study are included in the article/supplementary material, further inquiries can be directed to the corresponding author.

## Author contributions

YT was in charge of the data analysis and writing. YW was responsible for language modification and writing. JW was in charge of data collection and entry. RZ was in charge of the statistical analyses. QL was responsible for the study design and verification of the statistical results. All authors designed this study, contributed to it, and approved the final manuscript.

## Funding

This project was funded by the Shandong Medical and Health Science and Technology Development Plan Project (2018WS296).

## Conflict of interest

The authors declare that the research was conducted in the absence of any commercial or financial relationships that could be construed as a potential conflict of interest.

## Publisher’s note

All claims expressed in this article are solely those of the authors and do not necessarily represent those of their affiliated organizations, or those of the publisher, the editors and the reviewers. Any product that may be evaluated in this article, or claim that may be made by its manufacturer, is not guaranteed or endorsed by the publisher.
